# Development of Biological Coating from Novel Halophilic Exopolysaccharide Exerting Shelf-Life-Prolonging and Biocontrol Actions for Post-Harvest Applications

**DOI:** 10.3390/molecules29030695

**Published:** 2024-02-02

**Authors:** Chandni Upadhyaya, Hiren Patel, Ishita Patel, Parth Ahir, Trushit Upadhyaya

**Affiliations:** 1 School of Sciences, P. P. Savani University, Surat 394125, Gujarat, India; 2 School of Agriculture, P. P. Savani University, Surat 394125, Gujarat, India; 3 Shree P. M. Patel Institute of Integrated M. Sc. in Biotechnology, Sardar Patel University, Anand 388001, Gujarat, India; 4 Shree P. M. Patel Institute of P. G. Studies in Research and Sciences, Sardar Patel University, Anand 388001, Gujarat, India; 5 Chandubhai S. Patel Institute of Technology, Charotar University of Science & Technology, Changa, Anand 388421, Gujarat, India; trushitupadhyaya.ec@charusat.ac.in

**Keywords:** marine exopolysaccharide, coating material, % disease incidence, antagonistic attributes

## Abstract

The literature presents the preserving effect of biological coatings developed from various microbial sources. However, the presented work exhibits its uniqueness in the utilization of halophilic exopolysaccharides as food coating material. Moreover, such extremophilic exopolysaccharides are more stable and economical production is possible. Consequently, the aim of the presented research was to develop a coating material from marine exopolysaccharide (EPS). The significant EPS producers having antagonistic attributes against selected phytopathogens were screened from different marine water and soil samples. TSIS01 isolate revealed the maximum antagonism well and EPS production was selected further and characterized as *Bacillus tequilensis* MS01 by 16S rRNA analysis. EPS production was optimized and deproteinized EPS was assessed for biophysical properties. High performance thin layer chromatography (HPTLC) analysis revealed that EPS was a heteropolymer of glucose, galactose, mannose, and glucuronic acid. Fourier transform infrared spectroscopy, X-ray diffraction, and UV-visible spectra validated the presence of determined sugars. It showed high stability at a wide range of temperatures, pH and incubation time, ≈1.63 × 10^6^ Da molecular weight, intermediate solubility index (48.2 ± 3.12%), low water holding capacity (12.4 ± 1.93%), and pseudoplastic rheologic shear-thinning comparable to xanthan gum. It revealed antimicrobial potential against human pathogens and antioxidants as well as anti-inflammatory potential. The biocontrol assay of EPS against phytopathogens revealed the highest activity against *Alternaria solani*. The EPS-coated and control tomato fruits were treated with *A. solani* suspension to check the % disease incidence, which revealed a significant (*p* < 0.001) decline compared to uncoated controls. Moreover, it revealed shelf-life prolonging action on tomatoes comparable to xanthan gum and higher than chitosan. Consequently, the presented marine EPS was elucidated as a potent coating material to mitigate post-harvest losses.

## 1. Introduction

Exopolysaccharides of microbial origin have become valuable biomolecules in the last decade due to their safety, biodegradability, lack of toxicity, eco-friendliness, and economic production-like properties [1]. Due to their features viz. antimicrobial, antioxidant, good flavor, gelling agent, drug carrier, surfactant, flocculating, emulsification etc., EPSs are utilized in a wide range of industries including food, paint, textile, medicine, pharmaceutical, and agriculture [2,3]. Microbial EPSs have widespread applicability and commercial value viz. dextran, xanthan gum, pullulan, curdlan, gellan, scleroglucan, bacterial cellulose, etc., and have been implemented in the food industry for food processing and preservation. However, the research on exploration and production of such biopolymers for getting better applicability, efficacy and economic range is necessary for getting newer products better than those already available in the market [4]. Numerous extremophilic organisms have been reported to produce biotechnologically important products including exopolysaccharides with elegant properties and industrial applications. The reason behind it is the fact that extremophiles can withstand harsh environments for which they develop unique adaptive mechanisms and as a part of this, such novel protective biopolymers are produced [5]. The halophilic bacteria have been investigated and proven as an economic source of exopolysaccharides having industrially important characters whose consequent applications were testified by various researchers and illustrated in Table 1. 

In the food industry, a wide range of treatments were employed to improve the post- harvest preservation and productivity of crops. However, the physicochemical methods utilize harsh conditions which reduce the nutritional value of food items. Moreover, chemical treatments are sometimes hazardous to human health and also leads to the emergence of resistant phytopathogens [18]. Moreover, chemicals have less degradability when accumulated in the environment and when used in food industry, as they contaminate soil and water via waste and effluent discharge. Consequently, sustained production and usage of biological materials for food protection, preservation and post- harvest applications is very crucial and has become a prime area of research.

Another good example of such EPS is gellan gum which has been proven as a potent gelling agent and used in jams and confectionery food items. EPSs act as an edible film or coating material and are a very good alternative for food preservation due to potential health benefits and antimicrobial properties attributed due to the ionic interaction with the microbial cell wall and consequent growth inhibition capacity [19]. Additionally, levan- and pullulan-like homopolysaccharides have recently been applied as food coating. Pullulan was obtained from the fungus *Aureoblastidium pullulans* and is commercially utilized widely as edible film and packaging material [19]. Other than that, it is utilized as food additive viz. for food preservation, flavor and as a texture enhancer. Ref. [20] found that the incorporation of plasticizer sorbitol to EPS (dextran) resulted in the betterment of its elasticity and tensile strength, which helped in developing more stable packaging films. The edible food coating film was developed from blends of chitosan derivatives and was reported to have significant antibacterial potential with high stability [21]. The edible biofilm developed from pullulan was incorporated with ZnO and Ag- nanoparticles as well as rosemary essential oil and deduced as highly effective against the foodborne pathogens viz. *E. coli*, *S. aureus*, *S. typhi*, etc. [20]. One such biofilm developed from xanthan gum via adding it in antioxidant compounds like cinnamic acid resulted in the protection of browning of coated fruits, which is due to action of such films as an oxygen barrier. The antioxidant compounds embedded in EPS act as free radical scavengers and avert oxidation, resulting in the browning of fresh fruits. The ideal food coating material should not negatively affect the taste, odor or texture of coated food and should be stable to withstand the environmental variables and storage conditions viz. temperature, humidity and prolonged nonsterile storage [22]. 

The research gap addressed in the current research aimed to obtain stable halophilic EPS that is potent to antagonize the growth of phytopathogens as well as suitable as a coating material for postharvest shelf-life prolonging action on fruits and vegetables. The halophilic EPSs are less explored in the food industry as an edible coating material. Moreover, extremophiles are natural over-producers of such biological secondary metabolites and the ability to withstand stress of such extremophilic metabolites is also higher. Therefore, the chances of obtaining sturdy polymers with economic and stable production are more likely to be achieved. 

## 2. Results and Discussion

### 2.1. Screening of EPS-Producing Halophiles from Marine Samples

**Figure 1:**
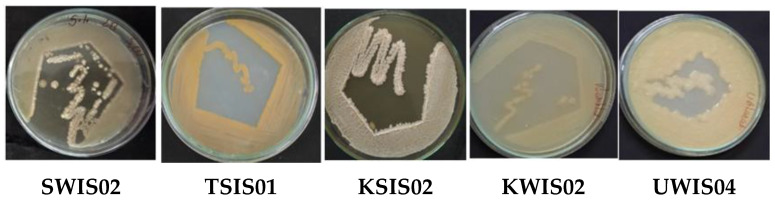
EPS producing halophiles selected after primary screening.

Halophilic microorganisms have been explored to obtain valuable and novel products with bioactive properties and industrial utilizations. The marine water and soil samples were analyzed to obtain halophiles with a high EPS production, along with the antagonistic effect on phytopathogens. A total of 22 different EPS producing halophilic bacteria were obtained whose EPS production potency was assessed for stable production. Out of 22, 5 revealed higher EPS production and were selected after primary screening, the outcome of which is represented in Table 2 and Figure 1. There was tremendous exploration of marine water, soil, and deep ocean sediments for the isolation of marine microbes potent to produce industrially important products such as exopolysaccharides. The stable and economic EPS producers viz. extremophiles have always been beneficial for large-scale production and application [23,24]. The halophiles have been proven as a highly valuable source of such secondary metabolites and are isolated by using salt-containing media [3,13].

**Figure 2:**
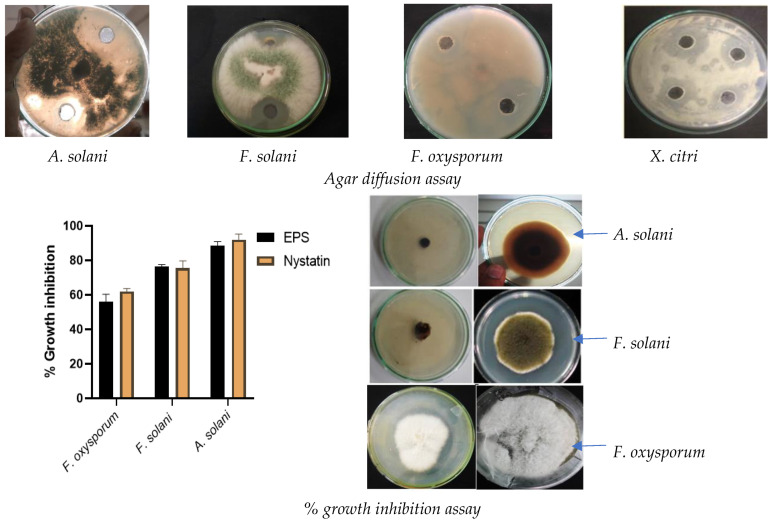
Antagonistic activity of EPS of TSIS01 by Agar diffusion assay and % growth inhibition by EPS against fungal phytopathogens. Five replicates were observed, and results were represented as mean ± SD which showed comparable outcome with positive control with no significant variation.

Secondary screening of selected isolates was obtained by checking the antagonistic effect of their EPSs on selected phytopathogens. The analysis revealed the growth inhibitory effect of selected halophilic EPSs on phytopathogens. The zone of inhibition was determined by the vernier calliper and presented in Table 2. The outcome revealed the highest zone of inhibition of EPS produced by TSIS01 isolate which was obtained from Tithal seashore soil. TSIS01 EPS showed growth inhibition comparable to streptomycin and nystatin positive controls with a variation of 6.16% to 10.16%. The % growth inhibition (% GI) of fungal phytopathogens in the presence of TSIS01 EPS was deduced by comparison with control plates which were devoid of EPS. Figure 2 illustrates the antimicrobial activity of TSIS01 EPS against phytopathogens whose ZOI (23.78 ± 1.25) and % GI (89.20 ± 2.30) revealed that EPS of TSIS01 presented the highest antagonistic effect against phytopathogenic fungus *Alternaria solani*. The mode of action exerted by EPS such as dextran on fungal pathogens demonstrated by [25] which revealed potential inhibition of hyphal growth via direct interaction that prevent fungal adhesion and limit nutrient availability.

On the basis of the growth inhibitory potential of exopolysaccharides against phytopathogens, TSIS01 was selected for further characterization whose EPS was also assessed thoroughly for stability, structural as well as biological properties, and suitability as a coating material. 

### 2.2. Characterization of Selected Isolate

The selected isolate was purified via repeated streaking on marine agar and the isolated colonies were pale yellow, mucoid, large, irregular, and smooth textured with slight elevation. The microscopic observation deduced that the isolated cells were gram-positive rods, motile, spore-forming, and capsule-forming bacteria. Moreover, the isolate can grow up to 50 °C temperature as well as 10% NaCl and thus, have a moderately thermo-halophilic in nature. 

#### 2.2.1. Biochemical Characterization

**Figure 3:**
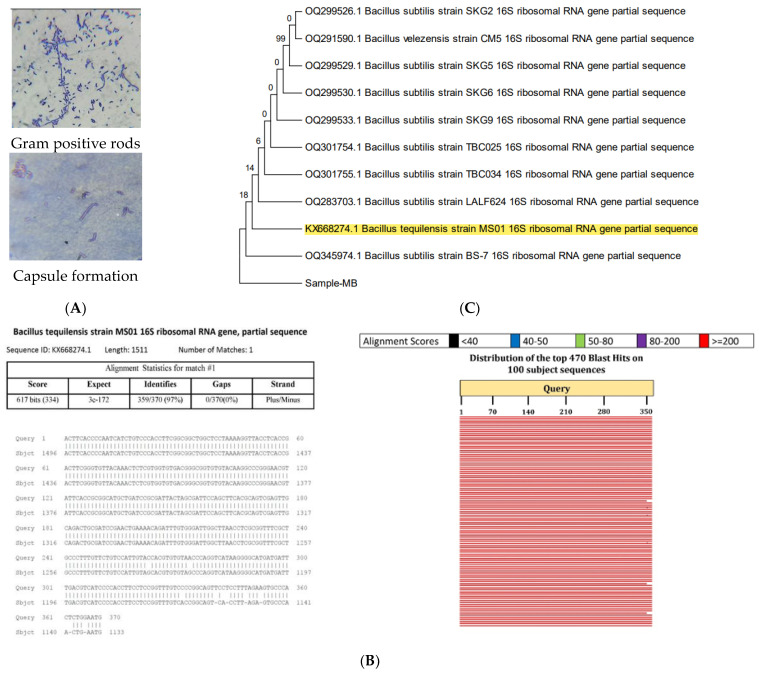
Characterization of TSIS01 by (**A**) microscopic (**B**) 16S rRNA sequence alignment, and (**C**) Phylogenetic tree revealed that the identified strain was *Bacillus tequilensis* MS01 that was highlighted.

TSIS01 gave positive tests for the triple sugar iron test (without black colored precipitates), VP test, alpha-amylase, citrate utilization test, cellulose hydrolysis, and catalase enzyme production tests whereas it gave a negative result for the indole test, MR test, oxidase, urease enzyme production, and nitrate reduction tests. The isolate can withstand temperatures up to 50 °C and grow up to 10% NaCl content. A similar biochemical analysis of Bacillus strains revealed that the isolate had high similarity with the *Bacillus tequilensis* strain [26]. The former documented a comparison of biochemical characters of the standard *Bacillus tequilensis* strain 10b^T^ and the presented isolate showed a high similarity and maximum possibility of the marine bacteria being *Bacillus tequilensis* which was further confirmed by molecular 16S rRNA analysis [27]. However, the isolate obtained by [28] was an endophyte and did not reveal capsule formation while the presented marine bacteria revealed capsule formation as illustrated in Figure 3A.

#### 2.2.2. Molecular Characterization

Molecular identification of TSIS01 by 16 S rRNA gene partial sequence analysis revealed a maximum (97.03%) similarity to *Bacillus tequilensis* strain MS01 (Accession: KX668274.1). The sequence alignment outcome and phylogenic relationship with other bacterial strains were illustrated in Figure 3 which revealed that *B. subtilis* strains were close relatives of *Bacillus tequilensis*. The maximum alignment hit score designated the isolate TSIS01 as *Bacillus tequilensis* MS01.

There were many marine bacilli species that have been testified as potent exopolysaccharide producers viz. *Bacillus thermodenitrificans*, *B. subtilis*, *B. licheniformis* strain B3-15 obtained in Italy from marine shallow-water vents of Vulcano island, as it reportedly produced EPS with unique antiviral activity [28]. 

### 2.3. Optimization of EPS Production

The various parameters affecting growth conditions and EPS production by a selected marine isolate TSIS01 were performed by a single factor at a time method and utilized for higher EPS production. The culture conditions at which lesser biomass with higher EPS production was obtained was considered optimum for EPS production. Each experiment was replicated a minimum of three times and results were represented as mean ± SD.

#### 2.3.1. Optimization of Culture Media

**Figure 4:**
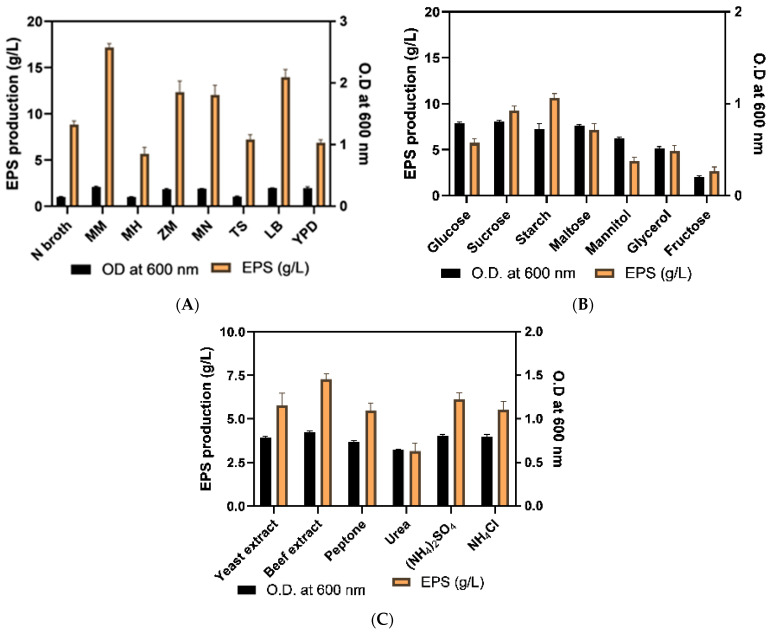
EPS production optimization by analysis of (**A**) different media, (**B**) carbon sources and (**C**) nitrogen sources (for incubation time 48 h). Each experimentation was performed in triplicate and results were represented as mean ± SD.

Exopolysaccharides are secondary metabolites produced by microbes and the scarcity of nutrients sometimes enhances exopolysaccharide production. According to [29], the choice of the most appropriate carbon source is a crucial practice for optimum EPS synthesis. Media optimization was obtained by the assessment of different media showing the highest EPS production along with optimization of each component of the media viz. C and N sources and their concentrations, P and K contents, Mg^+2^ and NaCl content. The outcome revealed the highest EPS production within minimal media (MM media) which was 17.17 ± 0.42 g/L after 5 d incubation (Figure 4A). The starch and beef extracts were deduced as the respective optimum C and N sources for maximum EPS synthesis by selected isolates (Figure 4B,C). Analogous EPS production in the starch-rich medium was documented by [30] in the case of lactic acid bacterial isolates obtained from gluten-free sourdough. However, [31] reported sucrose as an optimum carbon source for better EPS production via *B. tequilensis*. Razack [32] obtained a parallel optimum production of EPS by *B. subtilis* using beef extract as a nitrogen source.

The concentration of minimal media components along with salt (NaCl) was optimized for higher EPS production whose concentration range was chosen by MM media composition and former articles [1]. The starch was identified as the most potent carbon source for EPS production whose concentration range utilized for production was 5–40 g/L. There was a rise in biomass (O. D. at 600 nm) with C and N contents, but it was not more significant during 48 h of incubation. However, a linear increase in EPS content was reported with an increase in starch content which became maximum at 25 g/L (2.5%) and declined with further increase. The reason behind the decline in EPS lies within the fact that the EPS is a secondary metabolite and during higher C-source availability cell growth by primary metabolism is followed at a higher rate than EPS production which leads to a lower production of EPS [3].

**Figure 5:**
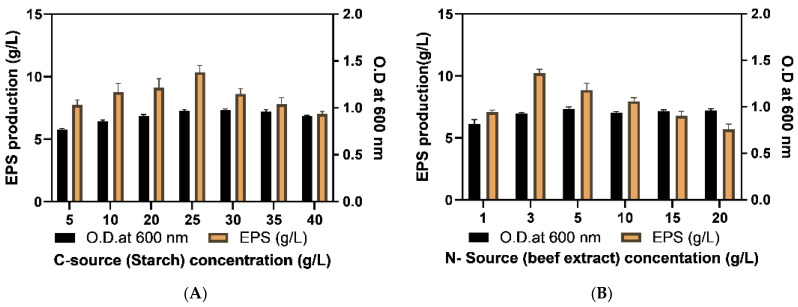

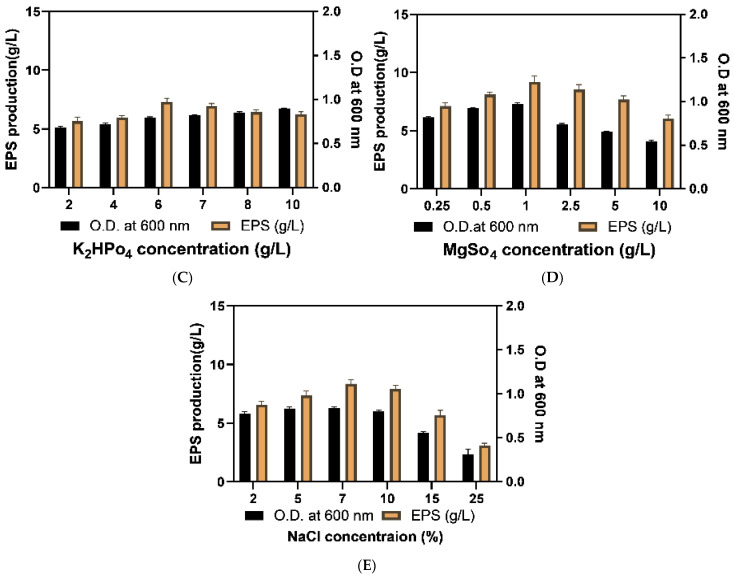
Optimization of concentration of minimal media (**A**) starch (C-source), (**B**) beef extract (N-source), (**C**) K_2_HPO_4_, (**D**) MgSO_4_ and (**E**) NaCl (%) for better EPS production at incubation time 48 h. Each experiment was performed in triplicate and results were represented as mean ± SD.

Similarly, the deduced potent nitrogen source was beef extract which was assessed in the range of 1–20 g/L. The increase in beef extract content increased cell mass. Although, the best EPS production was reported at 3 g/L (0.3%) after which EPS production declined significantly (Figure 5A,B). Wang et al. (2018) obtained similar highest production of exopolymers via *Bacillus thuringiensis* 27 in broth substituted with beef extract at a concentration of 3 g/L. Hence, optimum C and N concentrations for EPS production were 25 g/L and 3 g/L, respectively, which showed that higher C/N ratio is beneficial for EPS production. The presented outcome was analogous to the former investigations which also documented that a higher C/N ratio is required for better EPS production [33].

**Figure 6:**
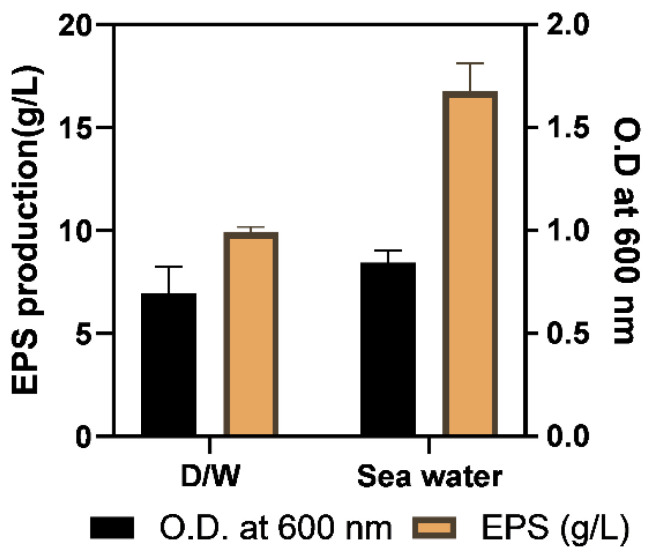
Effect of water (D/W and seawater) on EPS production. Each experiment was performed in triplicate and results were represented as mean ± SD.

The K_2_HPO_4_ was assessed for better EPS production and taken in a range of 2–10 g/L. There was an increase in EPS production with K content which reached a maximum of 6 g/L (Figure 5C). However, a slight decline in O.D (cell density) and EPS content with a further increase in K_2_HPO_4_ content was reported. An optimal level of K^+^ promotes osmoregulation the and maintenance of the electric potential of bacterial cell membranes, but a high K content slows down cell multiplication [34]. Along with N, P, K, and carbon source, Mg was also optimized due to its inevitable importance in cell metabolism [35]. The range of MgSO_4_ content chosen was 0.25–10 g/L. The variation in O.D. (cell density) and EPS production with MgSO_4_ content showed the highest EPS content at 1 g/L MgSO_4_ that was chosen for production media. According to [35], MgSO_4_ and KCl were reported to have a significant impact on polymer synthesis. For halophilic organisms, the NaCl content was a very important parameter for secondary metabolite production and therefore was assessed in the present study to evaluate its effect on EPS production. The range chosen for testing was 2–25 g/L. There was no significant change in biomass up to 10 g/L because the isolate was moderately halophilic (can withstand 5–15 g/L NaCl) and thus, metabolized NaCl at a uniform rate. Yet, the cell division rate significantly declined at 15 g/L and 25 g/L NaCl. The EPS content was highest at 7% NaCl as depicted in Figure 5E. The former analysis of EPS production by moderate halophiles also revealed the significant effect of NaCl on EPS production which was highest at 10% NaCl [1]. Further analysis of media preparation using distilled water and sterilized marine seawater revealed that sterilized seawater media significantly (*p* < 0.01) increased the EPS production after incubation of 48 h which is illustrated in Figure 6. Ref. [36] also investigated EPS production using M1 media prepared in sterilized seawater to maximize the polymer production by marine bacteria that exemplified the effect of complex salt composition of sea water which affect metabolism and thus enhance EPS production. 

#### 2.3.2. Optimization of Physical Growth Parameters

**Figure 7:**
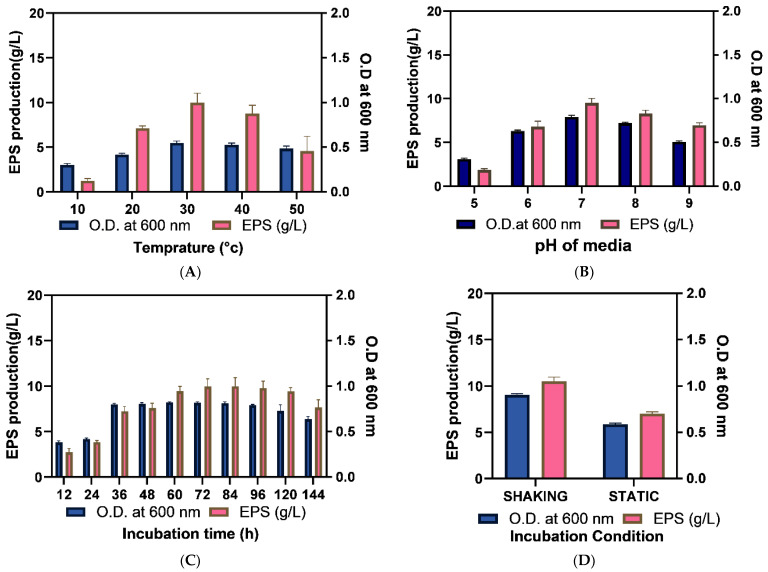
Optimization of physical parameters (**A**)Temperature, (**B**) pH, (**C**) Incubation time, and (**D**) shaking and static incubation conditions for higher EPS production. Each experiment was performed in triplicate and results were represented as mean ± SD.

The optimization of culture conditions is crucial to obtain a high microbial product yield. Consequently, temperature, pH, incubation time, and static or shaking conditions like parameters were evaluated in this analysis. The selected halophilic isolate can withstand temperatures up to 50 °C and results in constant biomass production from 30 to 50 °C. Temperature was deduced as one of the significant factors influencing EPS production and the optimum temperature reported was 30 °C at which EPS production was the highest (9.99 ± 1.04 g/L). It is noteworthy that the optimum growth temperature for a specific isolate may not match the temperature required for effective EPS synthesis [37]. Consequently, the enzymatic activity of different strain-specific glycosidases depends on the critical temperature at which the maximum synthesis of polymers is obtained at a high rate, and it has been widely documented that significant EPS synthesis was reported at a temperature lower than required for optimum growth of the isolate [30]. Ref. [30] reported a similar optimum temperature of 37 °C for exopolysaccharide production by *B. licheniformis* KS-17 obtained from kimchi. The pH optimization of media showed the highest EPS production at pH 7 (Figure 7A,B). The obtained outcome is parallel to the findings of [38] and [39] who reported that neutral pH was optimum for EPS production by respective *Pseudomonas aeruginosa* and *Bacillus tequilensis* PS 21 isolates.

For harvesting biomass from the production batch, and to recover the highest quantity of EPS, the optimum incubation time was deduced. Previous research observed that the extended incubation or fermentation period decreased EPS production due to the action of glycohydrolases [4]. The outcome showed that maximum EPS production was achieved upon 72 h of incubation, which remained constant (10.33 ± 0.68 g/L) up to 96 h however after that it declined. Thus, EPS can be recovered between 72 to 96 h periods as illustrated in Figure 7C. It is noteworthy that EPS reduction upon over-fermentation is dependent on the type of producer strains and physicochemical culture conditions. Assessment of incubation conditions viz. static and shaking conditions revealed that shaking at constant speed increased the EPS production. Consequently, the even distribution of cells and media components resulted in enhanced cell density and EPS production by 52.54% and 50.57%, respectively (Figure 7D). 

For getting higher EPS production, optimized condition for each parameter was determined and mentioned in Table 3.

#### 2.3.3. Assessment of the Efficacy of Optimization

**Figure 8:**
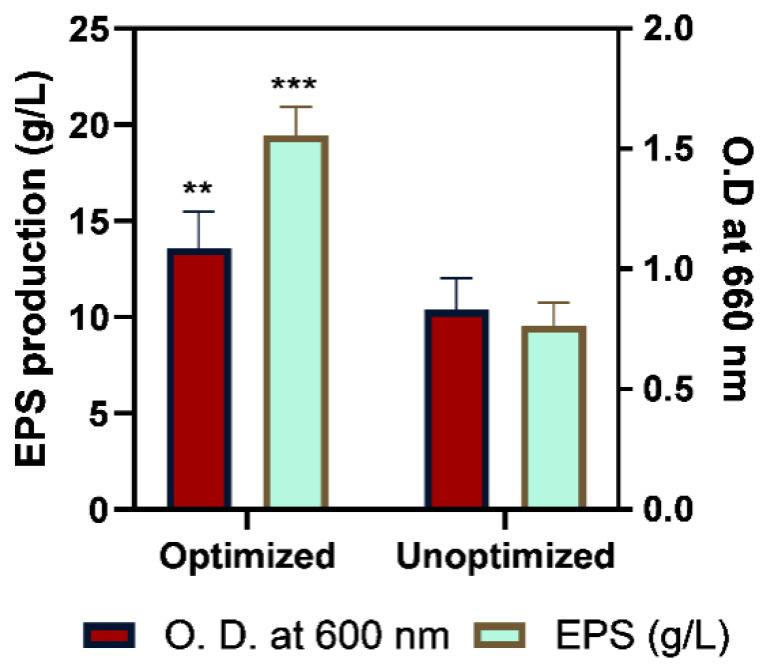
Comparison between optimized and unoptimized culture conditions of EPS production for checking the effectiveness of optimization. The significant variation in O.D. at 600 nm and EPS production is denoted as ** for *p* < 0.01 and *** for *p* < 0.001.

The effectiveness of the optimization procedure was evaluated by comparison of EPS production with unoptimized minimal media and culture conditions viz. 37 °C temperature, pH 7.0, incubation time 72 h, and shaking condition. The optimized conditions mentioned in Table 3, significantly (*p* < 0.001) enhanced EPS production by 188.69% more than the unoptimized culture conditions, as illustrated in Figure 8. Consequently, the obtained outcome would be utilized for scale-up by selecting the most significant parameters for further exploration of response surface methodology and application of the most optimum culture conditions in bioreactor based batch production. The optimum EPS production of 19.47 ± 1.45 g/L was reported after physicochemical optimization, which was better than the formerly reported EPS quantity of *B. tequilensis* strains. Sutthi [40] reported that *B. tequilensis* PS21 strain produced 15 g/L EPS in optimized conditions. However, [39] enhanced the EPS production up to 24 g/L by culturing the *B. tequilensis* PS21 in riceberry broken rice, and soybean meal broth. Suitable optimization enhanced EPS synthesis in many bacillus strains viz. *Bacillus licheniformis* in optimized media produced the highest EPS yield of 48.57 g/L [41]. 

#### 2.3.4. Determination of Growth Pattern and Phase of EPS Production for Harvesting

**Figure 9:**
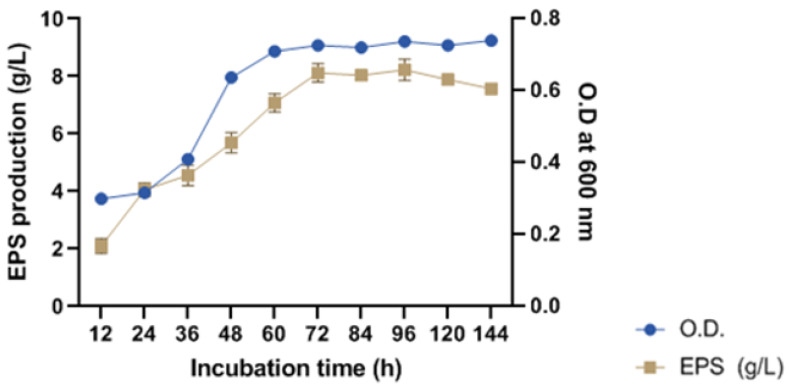
Determination of phase of growth cycle ofTSIS01 for EPS production.

The periodic evaluation of biomass density at 12 h intervals gave an idea about the growth pattern of the isolate which showed the exponential phase lasted from 12 h to 60 h followed by a stationary phase up to a 144 h time point. The EPS production was started in the exponential phase and maximized at a 72 h time point which lies within the stationary phase. A former investigation of *B. tequilensis* showed the maximum EPS production in the exponential phase [42]. The accumulation of EPS in the stationary phase was deduced as suitable for the harvesting of cells following the recovery of EPS from the production batch. However, in the late stationary phase, EPS content declined after 96 h because nutrients of media depleted with the increase in cell mass which resulted in the utilization of EPS as a carbohydrate source. The outcome obtained was similar to [43] who reported similar maximum EPS accumulation in the stationary phase which was also initiated in the exponential phase. Figure 9 illustrated the outcome which showed EPS production was in synchronization with cell growth and biomass density which is analogous to the majority of reports [44]. However, according to some findings, growth independent EPS synthesis which continue even at the stationary phase when cell division and biomass is limited. In the presented findings, the similar accumulation of EPS was reported during early and mid-stationary phase which was due to extracellular release and activity of glycosidases whose function is independent of the viability and growth of cells [41]. 

### 2.4. Characterization of EPS

The produced EPS was lyophilized and stored at −20 °C for further characterization viz. stability, composition, and bioactive properties for evaluating its potential for targeted application viz. as a food coating material in presented research. The main limitation of the utilization of such microbial polymers at the industrial scale is their stability.

#### 2.4.1. Assessment of Stability of Produced EPS

**Figure 10:**
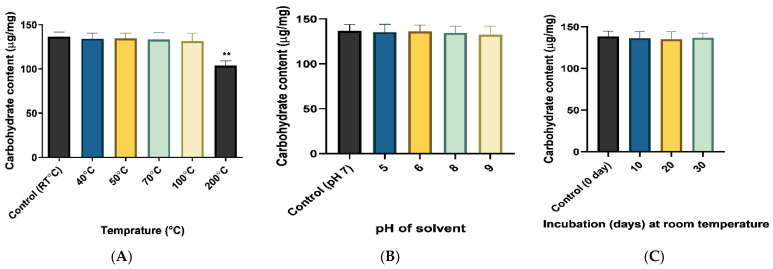
Analysis of EPS stability at different (**A**) Temperatures, (**B**) pH, and (**C**) Incubation time. The significant reduction in carbohydrate content was recorded at 200 °C (** *p* < 0.01) temperature as compared to room temperature.

The stability of EPS was evaluated by checking carbohydrate content at extreme temperatures, pH, and incubation period which was compared with the controls viz. 4 °C temperature, neutral pH, and 0-day incubation time (same day of EPS extraction and purification) while a notable reduction in carbohydrate content depicted the instability of EPS (Figure 10). There was no significant (*p* < 0.05) reduction in C content up to 100 °C compared to control EPS while it was significantly reduced at 200 °C which revealed that the obtained EPS was stable enough up to 100 °C. Moreover, there was no significant variation in carbohydrate content for the selected range of pH. The absolute stability at room temperature was reported after 30 days of incubation time as no reduction in carbohydrate content as compared to 0 d. Consequently, the obtained EPS was reported as stable enough to be used as a coating material. A similar stability check of exopolysaccharides was performed by [45]. 

#### 2.4.2. Determination of the Composition of EPS

##### Biochemical Composition and Spectral Analysis of EPS

**Figure 11:**
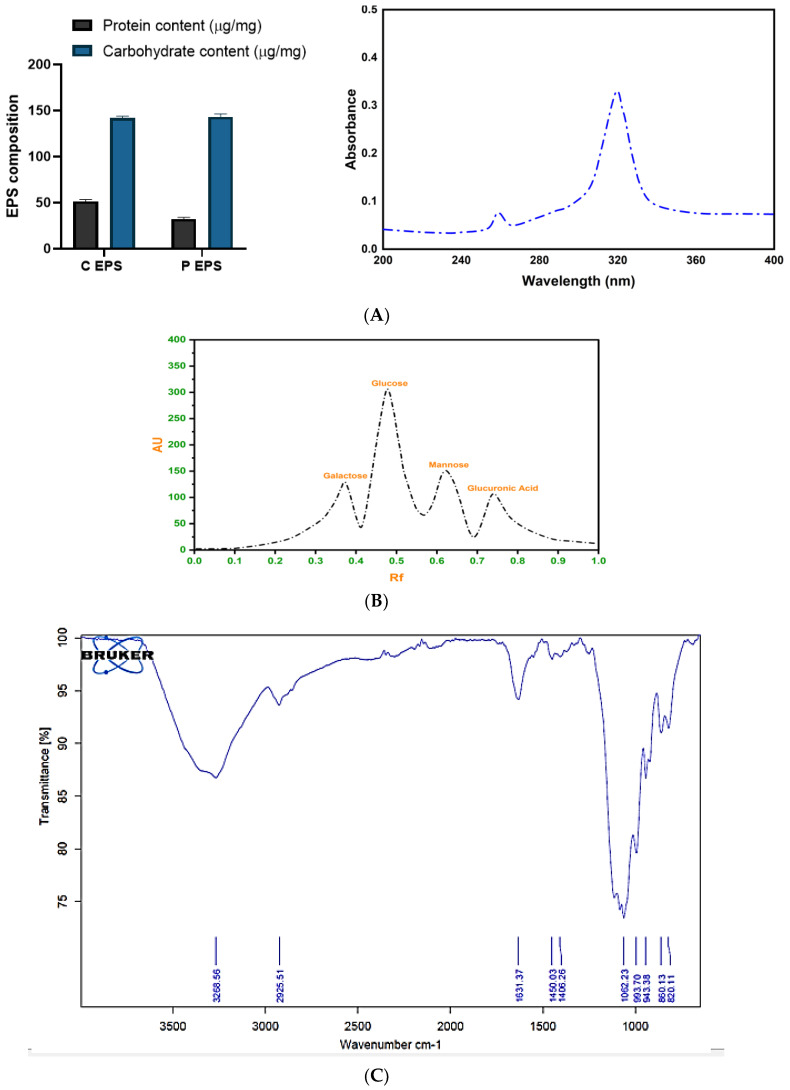
Analysis of the composition of EPS by (**A**) biochemical and spectral study, (**B**) HPTLC assay, and (**C**) FTIR analysis.

The composition of crude and deproteinized exopolysaccharides was assessed by biochemical quantification of carbohydrate and protein contents. The crude EPS comprises a high quantity of carbohydrates (141.70 ± 2.05 µg/mg) and protein (51.44 ± 1.89 µg/mg). Former reports documented that halophilic EPS contains higher protein levels which was reflected in the presented outcome. The deproteinized EPS comprised a reduced level of protein and slightly concentrated carbohydrates (Figure 11A). The UV-visible spectral analysis of EPS revealed the maximum absorbance (λmax) at 322 nm. The analogous polymeric peak showing EPS levan was obtained at 320 nm [46]. The peak was obtained at 260 nm showed the presence of proteins and supported the fact that the halophilic EPSs are rich in protein. According to a former report, the presence of protein in *B. licheniformis* EPS was confirmed by obtaining a peak at 260 nm [38]. Consequently, partial purification and the presence of a small quantity of protein provide structural support and stability of EPS. 

##### HPTLC Analysis

The acid-hydrolyzed sample of EPS resolved as four spots on the HPTLC plate and when read on the TLC reader identified as glucose, galactose, mannose, and glucuronic acid. The resulting well-separated sugar spots with retention force (Rf) 0.56, 0.58, 0.63, 0.71 were recognized as glucose (67.3%), galactose (19.5%), mannose (15.7%), and glucuronic acid (8.5%), respectively, compared with standard sugars. Thus, EPS was deduced as a heteropolymer in nature (Figure 11B). Former characterization and composition analysis of *B. tequilensis* EPS which were isolated from different locations revealed the strain-specific variation in the composition of EPS. However, a similar composition of EPS was produced by the halophilic bacteria *Halomonas maura* comprised of glucuronic acid, glucose, galactose, and mannose monomers [47]. Exopolysaccharides produced by marine bacteria were reported to contain uronic acid sugar monomers viz. glucuronic and galacturonic acid which were reported to possess skin and bone-restoring properties and are important in cosmetics and medicines [48]. Presented EPS contained such valuable acid sugar that made it a potential ingredient to be used in the food industry. 

##### FTIR Analysis

The FTIR analysis revealed that purified EPS possessed hydroxyl, carbonyl, and carboxyl-like reactive groups, as illustrated in Figure 11C. There was a broad strong peak obtained at 3268.55 cm^−1^ which indicated O-H or N-H stretch and the presence of amine, amide, or carboxylic acid [49]. The stretching vibration at 1631.37 cm^−1^ confirmed the presence of carboxylate bonds [50]. Moreover, EPS gave a specific band in the region of 1200–1000 cm^−1^ exemplified with coinciding stretching vibrations of the (C-O-H) side group and (C-O-C) glycosidic bond. Other than that, EPS having absorption at 994–820 cm^−1^ provided evidence for the α-glycosidic bond configuration The specific band of 1062.23 cm^−1^ designated the possible flexible α glycosidic bond and the band near 820.11 cm^−1^ indicated the presence of α- mannose units [51,52]. The absorption band near 1450 cm^−1^ was attributed to the bound water. Similar reactive functional groups and glycosidic linkages were reported in the EPS of marine *B. licheniformis* MS3 [53].

### 2.5. XRD, Molecular Weight and Rheological Behavior Analysis

**Figure 12:**
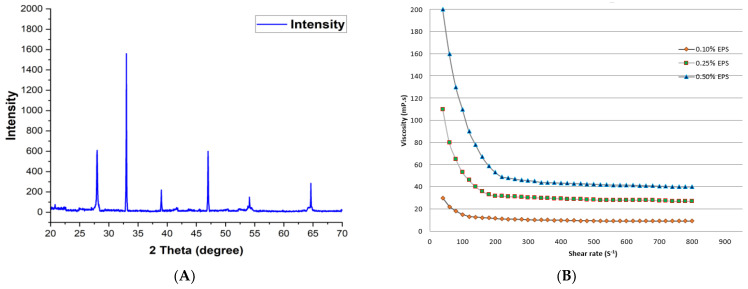
Analysis of EPS properties viz. (**A**) XRD analysis, (**B**) rheological behavior.

Phase identification of EPS and similar materials is widely obtained using the XRD technique. XRD of obtained EPS exhibited the representative diffraction peaks at 27.3°, 33.0°, 38.2°, 47.2° and 65.0° with respective d—spacing of 1.43°A, 1.21°A, 1.06°A, 0.895°A and 0.725°A. The XRD pattern demonstrated the amorphous nature of EPS with CI_xrd_ (crystallinity index) of 0.357 which is illustrated in Figure 12A. The crystallinity of EPS was deduced by taking the ratio of sharp narrow diffraction peaks and broad peaks and 35.7% crystalline domains improve the strength and functionality of polymer over wide-ranging temperatures (Shimazu, Miyazaki, and Ikeda, 2000). 

The calculated average molecular weight for the selected concentration 0.01 g/L was ≈1.63 × 10^3^ Da. 

The initial viscosity of produced EPS at room temperature was 198.5 mPa·s. The constant decline in viscosity with an increase in shear rate (0–800 s^−1^) exhibited the shear thinning conduct of EPS whose 2.5 g/L, 5 g/L, and 10 g/L concentrations were evaluated at room temperature (27 ± 2 °C) and revealed a similar flow pattern. Moreover, at high shear regions, the viscosity declined at a constant rate but upon elimination of shear strain, the polymer regained its initial viscosity which reflected the pseudoplastic nature of EPS. The reported rise in viscosity in a concentration-dependent manner revealed intermolecular entanglement of the polymer. An increase in shear rate leads to uncoiling and incomplete alignment of the polymer which decreases the viscosity. Analogous shear thinning was obtained by [54] in the case of a polymeric solution of *Lactobacillus sake*. The obtained pseudoplastic nature of produced EPS confirmed the suitability of its usage as a coating material.

### 2.6. Water Holding Capacity and Water Solubility Index of EPS

The produced exopolysaccharide was evaluated for water holding and solubility properties which are the critical parameters of food coating material for preservation [55]. Obtained EPS was soluble in water whose solubility index was 19.7% whilst the water holding capacity was 18.5% (18.5 g water/100 g EPS). The presented outcome showed low solubility and WHC comparable to xanthan gum (19.2 g/100 g) which helps to retain the water and prevents shrinking as well as damage to the coated material due to water loss. Similar outcomes were documented by [4] in which microbial EPSs viz. gellan, pullulan, and bacterial cellulose whose water-holding capacity was high that help to maintain the freshness of coated food. The edible coating or films comprising lower WHC and stability intern treated or incorporated with the stabilizers which increase the % WHC viz. corn starch increase the % WHC of edible film by 52.97% [56]. However, in the presented research such incorporation of stabilizers was not evaluated and can be explored in the next phase of experiments.

### 2.7. Evaluation of Bioactive Properties of EPS

#### 2.7.1. Antimicrobial Activity

**Figure 13:**
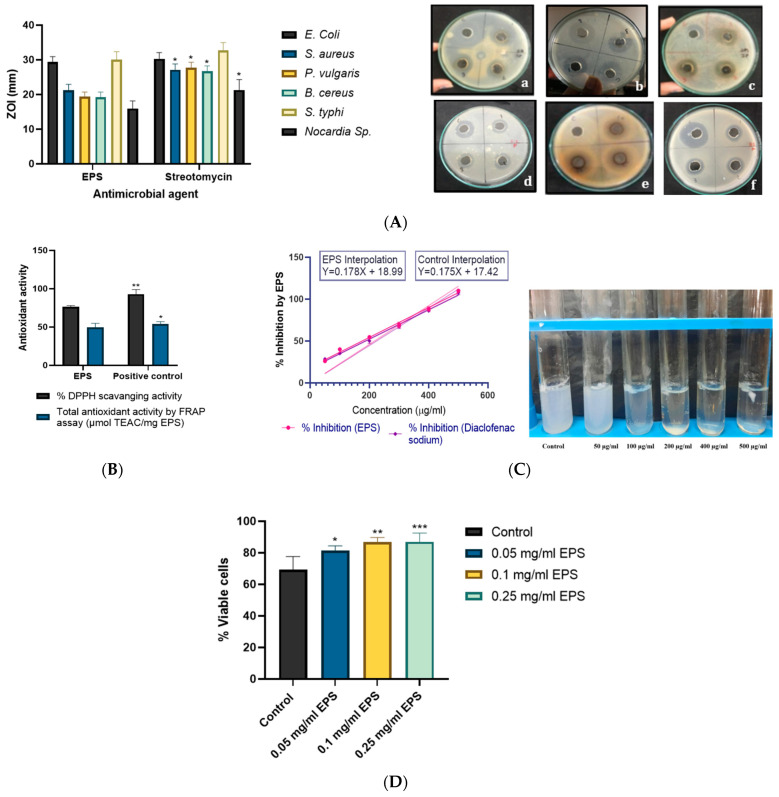
Analysis of biological activities viz. (**A**) antimicrobial activity against (a) *E.coli*, (b) *S. aureus*, (c) *P. valgaris*, (d) *B. Cereus*, (e) *S. typhi*, (f) *Nocardia* sp., (**B**) antioxidant, and (**C**) anti-inflammatory actions of EPS (**D**) Cytotoxic activity assay. % viable cells are higher in EPS inoculated tubes in concentration dependent manner compared to control tubes and extent of variation is denoted as * for *p* < 0.05, ** for *p* < 0.01, *** for *p* < 0.001.

Microbial polymers have been extensively utilized in the food industry as gelling agents, thickeners, emulsifiers, preservatives and coating material with different bioactive properties viz. antimicrobial, antioxidant, shelf life prolongation, antibrowning potential [57]. The antimicrobial activity of exopolysaccharides against six clinically important human pathogens was evaluated and the outcome revealed that it can govern growth inhibition of all six pathogens. The highest growth inhibition was obtained in the case of *E. coli* (29.36 ± 1.60 mm) and *Salmonella typhi* (30.00 ± 2.35 mm). However, the least inhibition was obtained in the case of *Nocardia sp*. (15.96 ± 2.17 mm) which reflected the resistance of *Nocardia* sp. to various antimicrobials [58]. It revealed intermediate growth inhibition of *Staphylococcus aureus*, *Bacillus cereus*, and *Proteus vulgaris* with respective zones of inhibition (ZOI) of 21.24 ± 1.69 mm, 19.18 ± 1.54 mm and 19.34 ± 1.38 mm. The antimicrobial activity of EPS was illustrated in Figure 13A which showed the comparable outcome of the antimicrobial activity of EPS with streptomycin (antimicrobial agent) in the case of *E. coli* and *S. typhi*. *E. coli* and *S. typhi* are gram-negative enteric bacteria that govern colon infection and as presented EPS can effectively antagonize their growth, coated food upon consumption provides additional benefits to consumers. Additionally, EPS also showcased inhibitory actions against selected gram-positive pathogens and thus can provide beneficial health effects which can be explored in the food industry as an effective coating material. Many reports illustrated similar antimicrobial activity of exopolysaccharides [59]. Rani [44] investigated the antimicrobial potential of heteropolymeric EPS of *Lactobacillus gasseri* against various pathogenic bacteria. The discriminating mechanism of actions is followed by different EPSs to exert antimicrobial action against the various pathogens viz. block the receptors required for interaction and cell-cell or cell-host communications, increase in competitiveness by auto aggregation, accumulation of secondary metabolites in media which adversely affect the pathogens [60], the interaction of functional groups with bacterial cells inhibit their growth [61], etc. 

#### 2.7.2. Antioxidant Activity of EPS

Oxidative stress can induce excess production of free radicals and reactive oxygen species which in turn induce cancer, aging, cardiovascular, and other diseases. The edible coating comprises antioxidant potential that can offer additional advantages in the form of mitigating oxidative stress and health benefits via consuming coated food products. Consequently, produced EPS was evaluated for its antioxidant activity by % DPPH radical scavenging activity and total antioxidant activity by FRAP assay via respective positive control of ascorbic acid and troxol. For comparative analysis, the concentrations of EPS and positive controls were kept constant at 4 mg/mL in the presented assay. For the selected concentration, EPS showed 76.28 ± 1.69% DPPH radical scavenging potential and 49.68 ± 5.19 μmol TEAC/mg EPS total antioxidant activity by FRAP assay (troxol equivalent) and showed a significant reduction of ferric-tripyridyltriazine to ferrous-tripyridyltriazine which was comparable to positive control Troxol. However, the % DPPH scavenging activity of EPS was 18.08% lesser than the ascorbic acid control (93.12 ± 5.87). The obtained results were comparable to the most popular exopolysaccharide of the food industry viz. xanthan gum [25,62] curdlan. Bomfim [50] reported an increment in the antioxidant potential of EPS in a concentration dependent manner. The reported outcome was better than the EPS produced by *Bacillus tequilensis* strains isolated from other sources viz. Luang-In [63] investigated EPS from *B. tequilensis* PS21 from Thai milk kefir that revealed 54.5% DPPH scavenging action and 34.9 µmol TEAC/mg EPS via FRAP assay which was lower than the obtained outcome. Yet another strain of *B. tequilensis* FR9 isolated from chicken gave 71.8% DPPH scavenging action at 4 mg/mL concentration [64]. Nevertheless, the presented marine *B. tequilensis* EPS exerted superior antioxidant potential which is advantageous for the exploration of the presented application as food coating.

#### 2.7.3. Anti-Inflammatory and Cytotoxic Activity

The in vitro anti-inflammatory assay was done by checking the heat protective effect of EPS after incorporating it in egg albumin which upon heating revealed the heat denaturation of protein and consequent rise in O. D. (at 660 nm) of the solution. The denatured protein turns the solution fuzzy whereas the heat-protected protein in the solution comprising an anti-inflammatory agent remains clear (Figure 13C). The % inhibition was calculated from the O.D of the test and control (Table 4) and revealed the anti-inflammatory potency of EPS. For comparison, diclofenac sodium was utilized as positive control which is a standard anti-inflammatory drug. The IC_50_ value is the content of the anti-inflammatory agent required to govern 50% inhibition of heat denaturation of the protein. The IC_50_ value of EPS and the control drug was determined by a standard plot of concentration (of anti-inflammatory agent) versus % inhibition (of heat denaturation) (Figure 13C). The deduced IC_50_ value of EPS was 174.21 µg/mL whereas the control drug was reported to have 186.17 µg/mL IC_50_. Consequently, EPS needs less concentration to govern 50% inhibition than the control drug and exerted higher anti-inflammatory potential which aids in the betterment of health upon consumption in the form of edible coating. To the best of the author’s knowledge, similar in vitro anti-inflammatory activity of microbial exopolysaccharides used in edible coating has not been reported.

Former investigations on the mode of anti-inflammatory action of EPS illustrated that, EPS can check interaction between antigenic determinants (viz. peptidoglycan and LPS of gram positive and negative bacteria or fungal proteins) and Toll like receptors (viz. TLR4) of inflammatory cells. EPS treatment also suppresses pro-inflammatory mediators, such as cyclooxygenase-2, tumor necrosis factor-α, interleukin-6, and interleukin-1β, and down regulates the expression of an inducible nitric oxide synthase responsible for oxidative stress. EPS also down regulates inducible phosphorylation and translocation of transcription factor NF-κB in the nucleus, which is involved in inflammatory responses. It also significantly down regulates phosphorylation of MAP Kinase and enhances the antioxidant marker expression (viz. NRF2 and HO-1 like protein expression).

It is common to assess the cytotoxic effect of microbial products to rule out any risk associated with health problems upon consumption. A similar analysis was performed for produced EPS by using 10-day-old chick embryo liver cells. In place of cytotoxicity, EPS exerted a positive effect on the cell viability of liver cells which was increased with an increase in concentration of EPS (Figure 13D. The highest cell viability was obtained in the case of 0.25 mg/mL EPS which was 87 ± 5.56% which was significantly (*p* < 0.013) higher than the control. None of the former reports showcased the cytotoxic property of *B. tequilensis* EPS on normal cells. However, few researchers found that microbial exopolysaccharides can exert a cytotoxic effect on cancer cells [65]. Consequently, the presented EPS was deduced as noncytotoxic and safe to be utilized in edible coating. A parallel cytotoxic potential of EPS was evaluated by application on gingival fibroblast cells which revealed a reduction in cell damage and was investigated by MTT assay [66]. Sutthi reported the beneficial effect of *Bacillus tequilensis* P21 on hematological parameters viz. RBC, WBC count, and hemoglobin protein of fish. 

### 2.8. Effect of EPS on the Shelf Life of Tomato Fruits

To investigate the effect of EPS coating on the shelf life of tomato fruits, firmness (N) and weight loss (%) of fruits were evaluated in a time-dependent manner and compared with uncoated negative control fruits and chitosan and xanthan gum-coated positive control fruits. 

#### 2.8.1. Firmness and Weight Loss (%) Analysis

**Figure 14:**
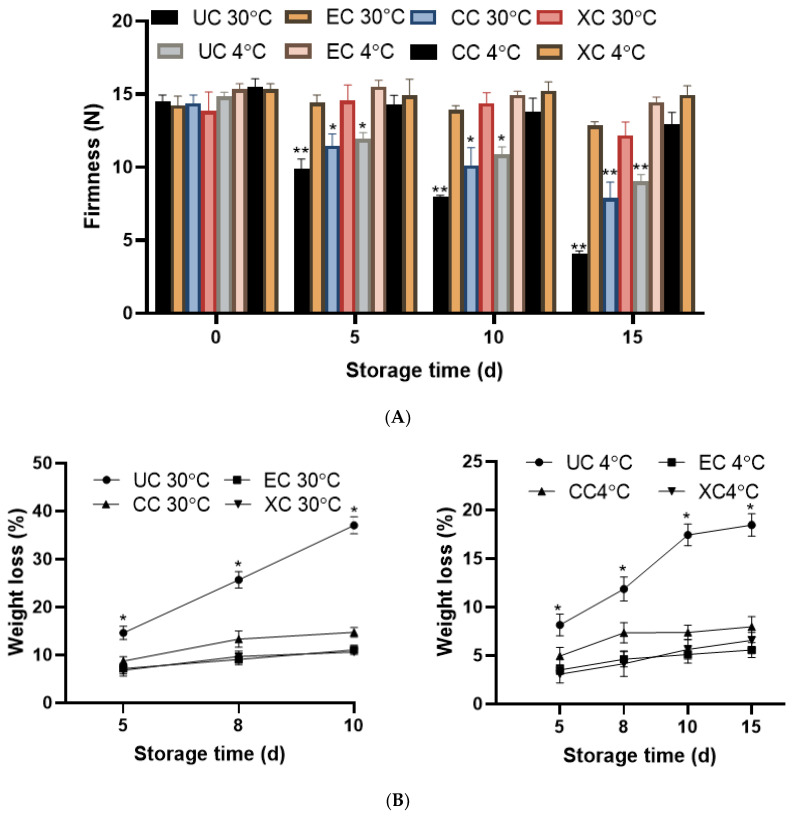
Effect of EPS coating on (**A**) firmness and (**B**) weight loss (%) of tomato fruits. Comparative statistical analysis revealed significant variation (*p* < 0.05) in chosen parameters for periodic incubation in case of control or uncoated fruits and marked as * superscript. * *p* < 0.05, ** *p* < 0.01.

The firmness of tomato fruits declined with prolonged storage. Although a faster decline was reported in the case of uncoated fruits (UC) at both 4 °C and 30 °C temperatures, it was more prominent at 30 °C than at 4 °C. The firmness of all groups of fruits at 5, 10, and 15 days storage times was compared with the 0 day which showed a non-significant reduction in the case of EPS-coated (EC) and positive control xanthan gum (XC)-coated fruits for both 30 °C and 4 °C temperatures and illustrated in Figure 14. Whilst chitosan-coated (CC) fruits were reported to show a significant (*p* < 0.01) reduction of firmness upon 15 days storage time compared to EC and XC. On the 10th day, the firmness of uncoated fruits was reduced by 50%, while the reduction was only 9.76% in EPS-coated fruits. Similarly, the reduction in firmness was even lesser in extent and was 5.67% at 4 °C in EPS-coated than uncoated (39.4%) tomatoes. Chitosan-coated fruits revealed a lesser decline in firmness at 4 °C and all-time points of storage than the 30 °C. Consequently, the presented EPS coating validated the positive effect on preservation of firmness at both selected temperatures whose effect was better than chitosan and comparable to xanthan gum. Softening of the fruits is an important indicator of fruit ripening and post-harvest storage which is performed by pectin degradation of the cell wall along with deterioration of intracellular material [67]. The major factor affecting fruit softening is temperature whose effect was also reflected in the presented research viz. at higher temperatures (30 °C) the firmness reduction was more than 4 °C. Better preservation at lower temperatures is due to the inhibition of enzymatic reactions of polygalacturonase and pectinesterase [27] involved in the softening of fruits. Accordingly, on the 15th day of storage time, the highest deterioration and fruit softening was reported in fruits kept at 30 °C. The retention of such softening of fruit tissue was reported by coating of fruits which reduced the respiration rate and enzymatic degradation and parallel to [68]. 

The related parameter of weight loss of fruits was assessed to appraise the efficacy of EPS coating compared to control groups. The outcome designated a raise in weight loss as time progressed which was the highest on the 10th day in fruits stored at 30 °C. Lowest weight loss (%) was reported in fruits coated with EPS (5.5%) and xanthan gum (6.5%) at 4 °C which was much lower than uncoated controls (18.46%). Accordingly, at 30 °C, in EC (11.05%) and XC (10.60%) groups of fruits, lesser weight loss was reported than the uncoated control (37.04%). The chitosan coating (CC) also revealed higher weight loss than presented EPS at 30 °C (14.72%) and 4 °C (8.50%). The significant reduction (*p* < 0.05) in weight loss of EPS-coated samples stored at 30 °C and 4 °C proved the efficacy of EPS coating in weight loss reduction that is illustrated in Figure 14. The major reasons behind the weight loss of fruits are water loss due to transpiration and vapour pressure which was slowed down by coating viz. EPS coating with hydrophobic components [69]. Along with transpiration, respiration, and water loss due to environmental and storage conditions are the prominent reasons for weight loss in fruits [70]. The possible effect of EPS in the reduction of weight loss is due to its action as a semipermeable barrier against oxygen, carbon dioxide, and moisture which resulted in a reduction in oxidation, respiration, and water loss from coated fruits. 

#### 2.8.2. Analysis of the Shelf Life of Tomato Fruits

The consumption quality of fruits was evaluated by considering two parameters viz. weight loss and firmness of the fruits. Consequently, in the presented study, the shelf life of fruits was estimated by kinetic models based on changes in these two parameters with time and different treatments and presented in Table 4 and Table 5. The outcome suggested significant modulation in both parameters (*p* < 0.05) between different treatments. According to calculated estimates (Table 4), the firmness parameter was discovered to fit in a second-order model for uncoated fruits at both temperatures and for all three coated groups (EC, CC, and XC) at 30 °C. However, at 4 °C, in all three coated groups, zero-order kinetic reactions were shown. For weight loss attributes, in all treatments, zero-order kinetics was followed. 

Table 5 represents the shelf life of tomatoes that showed the most profound effect of the weight loss attribute on it as in all treatments, conventional threshold was attained in a lesser period than firmness. In the case of uncoated fruits, the assessed shelf-life was 5 d at the storage condition of 4 °C and 3 d at 30 °C. Though the coating with EPS and standard chitosan and xanthan gum reduced the rate of weight loss at both temperatures, halophilic EPS was reported to have the highest shelf life prolongation by 12 d and 10 d, respectively, for 4 °C and 30 °C as compared to uncoated controls. EPS coating reduced the rate of weight loss comparable to xanthan gum which was reported to prolong the shelf life by 13 d and 9 d for 4 °C and 30 °C respectively. The positive control chitosan coating was reported to have a lesser shelf life-prolonging effect on tomatoes than xanthan gum as well as presented EPS. A similar shelf-life-prolonging effect of coating on cherry tomatoes was documented by [68] who experimented with exopolysaccharides produced by *Lactobacillus plantarum*. The outcome also exemplified the advantageous effect of EPS coating on the firmness of fruits, as they remained firm 22 d longer at 4 °C and 17 d more at 30 °C than the uncoated fractions. Consequently, presented EPS was reported as a suitable coating material for the shelf-life prolongation of fruits and a potent commercial agent to be used in the post-harvest application of the food industry.

### 2.9. Effect of EPS on the % Disease Incidence

**Figure 15:**
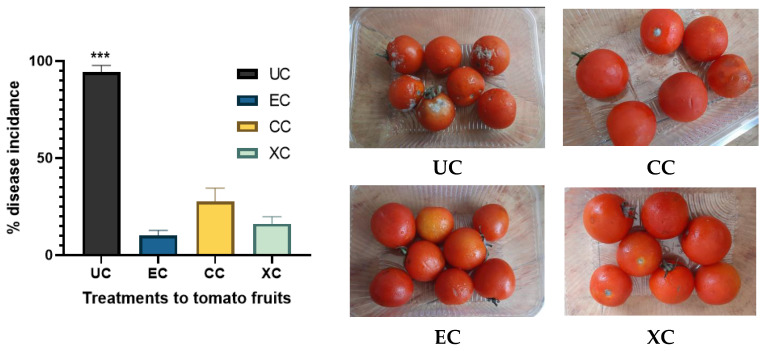
Analysis of % disease incidence in uncoated (UC), EPS-coated (EC), chitosan (CC) and xanthan gum (XC)-coated tomato fruits after treatment of phytopathogenic fungus *A. solani*. The highest variation reported in uncoated (UC) tomatoes than coated fruits (*p* < 0.001) and denoted as *** superscript.

% disease incidence analysis is a routine analysis for checking the efficacy of biocontrol agents against phytopathogenic infections. The effect of EPS coating on % disease incidence in tomato fruits was analyzed by further treating the fruits with the phytopathogenic fungus *Alternaria solani* that governs early blight disease in fruits. The outcome was compared with uncoated and positive controls viz. xanthan gum and chitosan-coated fruits and is illustrated in Figure 15. There was a significant reduction of 89.38% in % disease incidence reported in the case of EPS-coated groups compared to uncoated fruits. Comparative analysis of EPS coating treatment with chitosan and xanthan gum revealed a respective 63.77% and 38.27% higher efficacy of EPS in terms of reduction in % disease incidence. Thus, presented halophilic EPS gave a better outcome than xanthan gum and chitosan which significantly reduced the phytopathogenic onset of *A. solani* and can be applied to mitigate post-harvest losses due to early blight disease in tomato fruits. Similar evaluation of disease incidence after treatment of phytopathogens was not formally reported to the best of the author’s knowledge. The effect of microbial exopolysaccharides on fruits and other food products have been studied by various researchers [68]. 

## 3. Material and Methods

### 3.1. Sample Collection

The marine soil and water samples were collected from various beaches and a marine lake (Sambhar Lake) of Gujarat and Rajasthan states in India as mentioned in Table 6. Sampling was performed in the summer season from April to June, 2022. The water sampling was performed at a uniform depth. The depth of water near the shore was deduced by using a thick string wrapped with a rock at its end and a 500 mL plastic bottle was used for the same; the average depth used was 150–200 cm for all water samples. The soil samples were collected in a randomized manner by forming sectors on beach land [71].

### 3.2. Screening of Marine Samples for EPS Producing Halophiles

Primary screening of 7 marine soil and water samples was performed by utilizing Zobell marine agar (HIMEDIA) media to isolate halophilic bacteria having mucoid colonies. The isolates were subcultured on N-agar plates to confirm their halophilic nature. The selected EPS producing halophiles were further evaluated for EPS production potency by using Zobell marine broth (HIMEDIA) inoculated with 5% NaCl, and 5% glucose (pH 7.0) and incubated at 30 ± 2 °C for 72 h. For selecting higher EPS producers, the inoculated (loopful of the isolated colony) liquid media was assessed for cell density via taking OD at 600 nm following centrifugation at 7000 rpm for 20 min and the supernatant was collected for EPS quantification. EPS was recovered by adding three volumes of acetone following lyophilization (BST-LY101, Bionics scientific technologies, Delhi, India) and quantified using a precision scale to check production capacity of obtained isolates. Consequent comparative analysis amongst halophilic isolates resulted in selection of higher EPS producers.

### 3.3. Selection of Halophile Based on Biocontrol Potential of EPS against Phytopathogens

The phytopathogens utilized in this research were *Alternaria solani*, *Fusarium oxysporum*, *Fusarium solani* and *Xanthomonas citri* which were pre-isolated, characterized and stored. The selected EPS higher producers were assessed further for the antagonistic attributes of produced EPS (2 mg/mL D/W) against the selected phytopathogens via the agar diffusion method. The PDA agar plates were inoculated with a 5 mm colony of phytopathogenic fungus in the center and nutrient agar plates were inoculated with *X. citri* inoculum in which 20 µL of EPS solution was inoculated within wells and allowed to diffuse via incubating at 30 ± 2 °C for 24 h. Streptomycin (1 mg/mL) and nystatin (0.5 mg/mL) were utilized as positive controls in respective *X. citri* and other fungal pathogens comprising plates. The growth inhibition was determined by determining the zone of inhibition in (mm). A similar incubation of 5 mm fungal phytopathogenic isolate in the presence of EPS (2 mg/mL) incorporated in growth media as well as uninoculated control plates was performed to check % growth inhibition of fungal pathogens which was determined as following equation:

(1) $$\%\;Growth\;inhibition=\left(\frac{C-T}{C}\right)\times 100$$

where C = radial growth of fungus in control plate and T = radial growth of fungus in test plates.

### 3.4. Characterization of Selected Isolate

Colony and biochemical characterization of the selected isolate was done by standard protocols along with checking salt, temperature, and pH tolerance. Molecular identification of the isolate was performed by DNA extraction from isolate cells using a DNA prep kit (Quiagen, New Delhi, India). PCR of the 16S rRNA gene was performed using universal primers8F (5′-AGAGTTTGATCCTGGCTCAG-3′) and 1492R (5′-CGG TTA CCT TGT TAC GAC TT-3′) as mentioned by [72]. PCR was performed using ABI Veriti PCR Machine (Applied Biosystems, Waltham, MA, USA) and standardized program viz. denaturation at 93 °C for 1.5 min; 30 denaturation cycles at 93 °C; 50 °C for annealing; 30 s extension at 72 °C following a final extension of 5 min at 72 °C. PCR amplicons were purified by column purification and the sequence was identified by primers 27F (5′-AGAGTTTGATCMTGGCTCAG-3′) and 1492R (5′-ACCTTGTTACGACTT-3′) at SLS Pvt ltd. Surat, Gujarat, India. The obtained sequence of 16S rRNA segment was assessed by BLAST, compared to bacterial 16S rRNA sequence data of the Gene bank and aligned to maximum identity score sequences using a multiple alignment tool. The evolutionary distances were computed via a Maximum Composite Likelihood method and a phylogenetic tree was made using a neighbor-joining method with 500 bootstrap replications available in the MEGA version 6.0.

### 3.5. Optimization of Culture Conditions for EPS Production 

EPS production by the selected isolate was optimized by one factor at a time methodology whose replicated outcome would be utilized for further scale up via central composite design (CCD) (Not included in this paper).

#### 3.5.1. Optimization of Media

The (1%) biomass of the selected isolate was inoculated within different media broths (1000 mL) viz. Nutrient (N- broth), Minimal media (MM), Mueller Hinton (MH), Zobell marine broth (ZM), Mannitol salt broth (MN), Tryptic soy broth (TS), Luria- Bertani (LB), Yeast potato dextrose (YPD) to determine cell-mass (O.D. at 600 nm) and EPS content (g/L) after 48 h culture on shaker at 30 ± 2 °C. The carbon sources analyzed for optimized EPS production were glucose, sucrose, maltose, mannitol, glycerol, fructose, and starch for which 5% of each carbon source was inoculated in broth. The identification of good nitrogen source was also done similarly by utilizing 1% yeast extract, beef extract, peptone, urea, ammonium sulphate and ammonium chloride in production media. After selecting a carbon source, its optimum concentration was deduced by utilizing 5, 10, 20, 25, 30, 35 and 40 g/L concentrations. Likewise, nitrogen source content utilized for optimization was 1, 3, 5, 10, 15, 20 g/L. The K_2_HPO_4_ content was optimized by taking 2, 4, 6, 8, 10 g/L, MgSO_4_ content was optimized by taking 0.25, 0.5, 1.0, 2.5, 5.0, 10 g/L and NaCl content assessed for optimization was 2, 5, 7, 10, 15, 25%. The sterilized sea water and distilled water were analyzed for better EPS production by applying all optimized media components [73].

#### 3.5.2. Optimization of Physical Parameters

The inoculated (inoculum 1% having OD 1 at 600 nm) production broth was incubated at 10, 20, 30, 40 and 50 °C for temperature optimization. The effect of pH on EPS production was evaluated by applying pH 5, 6, 7, 8 and 9 in media. The effect of static and shaking (120 rpm) conditions was applied for EPS production [73]. 

### 3.6. Bacterial Growth Curve, Production, and Extraction of EPS

The isolated marine culture was inoculated in 200 mL of optimized production media filled in 500 mL Erlenmeyer flasks which were incubated on shaker at 140 rpm for 5 days at 30 ± 2 °C. The control unoptimized minimal media containing flasks were also inoculated and incubated similarly to deduce the effectiveness of optimization treatment. The uniform pH of 7.0 was adjusted (by 1 N NaOH) in both optimized and unoptimized media. Aliquot samples of 10 mL were withdrawn at 6 h regular intervals to determine the growth pattern (A600 nm) of the selected isolate. After deducing the stationary phase, the bacterial cells were harvested by centrifugation of broth at 10,000 rpm for 30 min at 4 °C, and the supernatant was further filtered by pressure filtrations via cellulose nitrate filters (Millipore filters, Bangalore, India) having 0.45 µm pore size. EPS extraction from the final filtrate was obtained by adding three volumes of ethanol following overnight incubation at −20 °C for complete precipitation. EPS precipitates were collected by centrifugation at 8000 rpm (at 4 °C) for 20 min. Collected EPS was lyophilized until complete dryness and stored at −20 °C for further use [74].

### 3.7. Deproteinization and Assessment of the Stability of EPS 

EPS was deproteinized by treating the EPS solution (10%) with TCA (20% *w*/*v*) overnight at 4 °C following centrifugation at 7000 rpm for 25 min to remove the protein precipitates. The collected supernatant was treated with Sewage reagent (Chlorophorm: butanol) (2:1), incubated for 15 min at room temperature, and centrifuged to separate proteins at the intermediate phase. Deproteinization steps were repeated three times. The pulled EPS fraction was dialyzed against MiliQ by using dialysis membranes of 12–14 KDa cut-off and the resulting partially purified EPS was used for stability and other characterization assays. The produced crude EPS was incubated overnight at temperatures ranging from (Room Temperature, 40, 50, 70, 100, and 200 °C), pH (5, 6, 7, 8, and 9), and for different time intervals (0, 10, 20 and 30 days) at room temperature. The stability of EPS (after incubation at the mentioned conditions) was assessed by determining its carbohydrate content via the phenol sulphuric acid method (absorbance was taken at 490 nm. Comparative reduction in carbohydrate content reflects the instability of EPS [75].

### 3.8. Characterization of EPS

#### 3.8.1. Composition, Spectrophotometric and FTIR Analysis

Crude and partially purified EPSs were assessed for their carbohydrate and protein contents by standard phenol sulphuric acid (against glucose standard) and Bradford method (against BSA standard), respectively [75]. EPSs (0.5 mg/mL D/W) were assessed spectrophotometrically by taking spectra from 200 to 600 nm (Manti Lab MT-137A, Maharashtra, India). The structural groups were determined by FTIR analysis. The 0.2 mg lyophilized powder of partially purified EPS mixed with 5 mg KBr and pelleted samples were assessed via Alpha II compact FTIR spectrophotometer. The IR spectra of 400–5000 cm^−1^ were discovered with a Hewlett Packard plotter. (Bruker, Mumbai, Maharashtra, India). 

#### 3.8.2. HPTLC Analysis for Sugar Composition of EPS

HPTLC analysis was performed to deduce the monomeric composition of EPSs for which partially purified EPS was acid hydrolyzed in 2M Trifluoroacetic acid (TFA) at 100 °C for 5 h. HPTLC of hydrolysate was performed by the modified methodology of [76]. The 5µL hydrolysate was applied on 30 × 20 cm HPTLC silica gel plate (Merck, Darmstadt, Germany) along with sugar standard viz. arabinose, glucose, galactose, sucrose, fructose, maltose, glycerol, glucuronic acid, galacturonic acid, xylose (Merck, Darmstadt, Germany) via HPTLC instrument (CAMAG, Muttenz, Switzerland) of Sophisticated Instrumentation Centre for Applied Research & Testing, Sardar Patel University, India. For sugar analysis, separation was performed using butanol: ethanol: H_2_O (5:3:2) mobile phase, and visible bands were obtained by application of ethanolic p- anisaldehyde + ethanolic conc. H_2_SO_4_ solution used as developing agents. The plate was dried, heated at 70 °C for 15 min, and put under white light for band visualization whose image was scanned and analyzed by the CAMAG visualizer system [76]. 

#### 3.8.3. XRD Analysis of EPS

The chemical nature and crystallinity of the EPS sample were determined by the X-ray powder diffractometer (Philips X’pert MPD, Almeno, The Netherlands). The lyophilized EPS sample was applied on a quartz substrate and scanned up to 10 mm whose continuous *X*-ray diffraction pattern was recorded at 25 °C. XRD characterization of EPS was done by slow scanning at different two theta angles ranging from 2° to 80° with a scan step time of 1 s. The value of d-spacing of diffracted X-rays was calculated for each value of *θ* according to Bragg’s law equation as follows:

(2) $$d=\frac{\lambda }{2\;sin\;\theta }\;$$

The Crystallinity index (CI_xrd_) of the sample was calculated from the normalized peak area related to the total scattering area (mentioned in the following equation).

(3) $${CI}_{xrd\;}=\frac{\sum A_{crystal}}{\sum A_{crystal}+\sum A_{amorphous}}\;$$

#### 3.8.4. Water Solubility Index (WSI) and Water Holding Capacity (WHC) of EPS

The WSI of EPS was determined by taking 100 mg of EPS in 3 mL of D/W and stirring at 35 °C till uniform solution (30 min). The suspension was centrifuged at 10,000 m rpm for 20 min, the wet residue was weighed, and the supernatant was dried at 100 °C until complete dryness and also weighed [77]. % WSI was deduced by the following formula:

(4) $$WSI=\frac{Weight\;of\;dry\;solid\;in\;supernatant}{Weight\;of\;dry\;sample}\times 100$$

WHC of EPS was determined by taking 200 mg EPS in 10 mL D/W and uniformly dispersed. The dispersed solution was centrifuged at 10,000 rpm for 30 min and the pellet was dropped on pre-weighed filter papers. The % WHC was calculated by the following formula:

(5) $$WHC=\frac{Total\;sample\;weight\;after\;water\;absoption}{Total\;dry\;sample\;weight}\times 100$$

#### 3.8.5. Rheological and Viscometric Behavior of EPS

The shear stress effect on EPS was assessed by viscometric examination using a stress-controlled rheometer (TA instruments, New Castle, DE, USA). The instrument was equipped with a double-gap cylinder assembly in which EPS samples of concentrations ranging from (0.05–2.0% (*w*/*v*)) were employed. Measurements of viscosity were performed over a wide range of shear rate (0.01 to 1000 s^−1^) and 25 °C. The dynamic measurements of strain sweep were carried out at 1 rad/s to deduce the linear viscoelastic regime of EPS solution by 0.1–200% strain [78].

#### 3.8.6. Intrinsic Viscosity and Molecular Weight Determination

The intrinsic viscosity of EPS was determined by the following equations utilizing the Ostwald viscometer. For viscosity analysis 0.01 g/mL EPS solution was prepared by using distilled water which was further utilized for determination of average molecular weight by the Mark Houwink Sakurada equation (Equation (9)) [79]. 

Relative viscosity:

(6) $${ƞ}_{rel}=\frac{t}{t_{0}}$$

Specific viscosity:

(7) $${\;ƞ}_{sp}=\frac{t}{t_{0}}-1$$

Intrinsic viscosity:

(8) $$[ƞ]=\frac{{ƞ}_{sp}}{C}$$

where t is the flow time of EPS solution, t_0_ is the flow time of solvent, C is concentration of the polymer.

The average molecular weight of EPS:

(9) $$[ƞ]=KM^{\alpha }$$

where K and α are predetermined empirical constants. 

### 3.9. Assessment of Bioactive Properties of EPS

#### 3.9.1. Antimicrobial Activity of EPS

The agar diffusion method was used to investigate the antibacterial activity of EPS. The bacterial pathogens were procured from microbiology department of institute and inoculum in broth was prepared by setting O.D. one at 600 nm (8 × 10^8^ cells). The EPS (5 mg/mL) was inoculated into the wells against different pathogenic test microorganisms inoculated (0.1 mL per plate) in media and incubated at 30 °C for 24 h. The zone of inhibition was deduced in millimeters. The test clinical pathogenic microorganisms viz. *E.coli*, *Bacillus subtilis*, *Bacillus cereus*, *Proteus valgaris*, *S. aureus*, *S. typhi* and *Nocardiopsis* sp. were used for antimicrobial activity [80].

#### 3.9.2. Antioxidant Activity by % DPPH Scavenging and FRAP Assay

% DPPH radical scavenging experiment was performed using standard ascorbic acid by standard methodology [81]. FRAP assay was performed using FRAP reagent (2.5 mL FeCl_3_ (20 mmol/L) + 2.5 mL TPTZ (2,4,6- tripyridyl-s-triazine) in 40 mmol/L of HCL + 30 mL of acetate buffer (300 mol/L), (pH 3.6) and assay methodology was refereed from Upadhyaya et al., 2022. The 250 μL EPS solution (3 mg/mL) was added to 1 mL fresh FRAP reagent. The mixer was incubated for 5 min and optical density was taken at 593 nm. The assay was performed by taking a standard Troxol solution which was evaluated as positive control.

#### 3.9.3. In-Vitro Anti-Inflammatory and Cytotoxic Assay

The egg albumin protein gets denatured upon heating whereas the heat protective effect of exopolysaccharide was evaluated as anti-inflammatory potential which was performed according to [46]. The test reaction mixture was prepared by 0.2 mL fresh hen’s egg albumin mixed with 2.8 mL of phosphate buffer saline and 2 mL of EPS dissolved in distilled water with different final concentrations viz. 31, 62.5, 125, 250, 500, and 1000 μg/mL. A control tube was added with D/W in place of EPS. As a positive control, the anti-inflammatory drug Diclofenac sodium of the same concentrations was evaluated. Both sets of tubes were heated for 7 min at 70 °C and evaluated for absorbance at 660 nm after cooling. The following equation was utilized to determine the % inhibition of protein denaturation [46]:

(10) $$\%\;inhibition=100\times \left(\frac{V_{t}}{V_{c}}-1\right)\;$$

where V_t_ is the O.D. of test and V_c_ is the O.D. of control at 660 nm

The cytotoxicity assay was performed based on the fact that damaged cells take up trypan blue dye whereas normal cells do not have that capability and do not turn blue upon incubation. If the sample compound is cytotoxic, it exerts a negative effect and in turn, damages the cells which take up more trypan blue and are counted as non-viable cells upon microscopic examination [82]. For the assay, a 10-day old chick embryo was dissected and liver tissue was homogenized in PBS buffer and incubated with EPS (0.05 mg/mL, 0.1 mg/mL, 0.25 mg/mL) and 1–2 drops of trypan blue dye for 15–20 min on a rocker at room temperature. The control tubes were inoculated with distilled water in place of EPS. The drops of cell homogenate from test and control tubes were microscopically examined for counting viable cells versus total cells and % cell viability was evaluated by following Equation (7) which was compared with control tubes (devoid of EPS).

(11) $$\%\;viable\;cells=\frac{Total\;no.of\;cells\;perml\;of\;aliquot}{Total\;no.of\;counted\;cells\;per\;ml\;of\;aliquot}\times 100$$

### 3.10. Preparation of EPS Coating Solution and Uniform Application on Tomato Fruits

For the preparation of the coating, a modified method of [68] was employed in which glycerol and oleic acid were used as a plasticizer and surfactant, respectively, that helped in the better adhesion of the coating material on the surface. For the preparation of emulsion, the emulsifying agent was used in the form of Tween 80 (Merck, Darmstadt, Germany). The hydrophobic phase was made by mixing 5% EPS (*w*/*v*) with 1% glycerol and the hydrophilic phase was developed by mixing 0.5% Tween 80 and 1% oleic acid. The hydrophilic phase was first added to 70 mL of water and solubilized via vortexing for 5 min followed by mixing the hydrophobic phase and making up a total volume of 100 mL. The coating solution was stored at 4 °C for 24 h to coat tomato fruits.

The true-to-type tomato fruits of the same variety were harvested from the greenhouse of the institute. The collected sample fruits were healthy, damage-free, and uniform in size as well as ripeness. The fruits were disinfected before coating by using 2% (*v*/*v*) sodium hypochlorite rinsing and repeated washing with distilled water, as well as air dried. Fruits were divided into four groups, treated with EPS coating solution, and negative as well as positive controls for comparative analysis as mentioned in Table 7.

The tomato fruits were coated uniformly by immersing in the coating solution for 60 s and allowed to dry for 1 h in a laminar airflow cabinet and incubated at two incubation conditions 4 °C and 30 °C. The shelved tomato fruits were assessed periodically for the effect of EPS on shelf life and % disease incidence.

### 3.11. Investigation of Shelf-Life-Prolonging Potential of EPS

The shelf-life of control and test tomato groups was calculated by considering two parameters viz. firmness and weight loss over the range of 15 d (0, 5, 10, 15 d) of incubation. The firmness of fruits was determined using a Shimadzu EZ food texturometer. The force required for penetration of fruit was measured by a blunt plunger having a 5 mm diameter from a penetration distance of 15 mm at 50 mm min^−1^ speed [68]. The maximum force (N) required to exert a tearing effect on fruit denotes firmness which was deduced for 5 replicates of each treatment.

The weight loss was calculated as % weight loss in reference to the initial weight of the sample tomato. An AUX220 weighing machine (±1 mg accuracy) (Shimadzu, Kyoto, Japan) was used to measure weight before and after the incubation period and calculated as follows:

(12) $$Weight\;loss=\frac{W_{i}-W_{f}}{W_{f}}\times 100$$

where $W_{i}$ = Weight of fruits at 0 d and $W_{f}$ = Weight of fruits after storage time t

The determination of these two parameters is followed by the determination of the reaction-order for each. Minimum time elapsed to reach the threshold value of one of these parameters and calculated as follows [83]:

(13) $$\frac{d[A]}{dt}=kA^{n}$$

where A = Parameter value to be calculated; n = Reaction-order; k = Reaction constant; t = time.

For each attribute, reaction order was evaluated based on Equation (13). The integrated model of each of the most common reactions affecting quality of food viz. zero, first and second order reactions whose evaluation equations are Equations (14)–(16), respectively. Thus, based on treatment (UC and EC) and storage conditions (30 °C and 4 °C), the best fit was selected for each parameter which is consistent with the maximum correlation coefficient (R^2^),

(14) $${\left[A\right]}_{t\;}=A_{0}-K_{e}t;n=0$$

(15) $${\left[A\right]}_{t\;}=A_{0}e^{-kt};\;n=1$$

(16) $$\frac{1}{\left[A\right]}=k_{e}t+\frac{1}{A_{0}}\;;n=2$$

where k is the reaction constant. The values of [A_0_] and k were determined and applied to the individual model, and from the deduced outcome, the shelf-life of fruits for each separate parameter was estimated whose threshold standard values considered for was maximum of 5% and minimum of 8 N [84] for weight loss and firmness, respectively. 

### 3.12. Assessment of the Effect of EPS Coating on Disease Incidence upon Treatment of A. solani

The EPS-coated and positive control as well as uncoated negative control fruits were treated with inoculum of phytopathogenic fungus *A. solani* which was prepared in potato dextrose broth. A total of 5 mL of inoculum was sprayed on all groups of tomatoes which were incubated at 20 ± 2 °C for 10 days and checked for onset of fungal colonization over fruits. The analysis was done by 5 replicates including 100 fruits for each treatment and % disease incidence [85] was deduced as follows:

(17) $$\%\;disease\;incidence=\frac{Total\;no.of\;fruits-diseased\;fruits}{Total\;fruits}\times 100$$

### 3.13. Statistical Analysis

All the analysis was performed as a minimum of three and maximum of five replicates and results were represented as mean ± SD. The statistical analysis of outcomes was performed using Graph pad Prism version 8.0 software using simple one-way and two-way ANOVA. Data analysis was evaluated for *p*-value and considered as significant if *p* < 0.05.

## 4. Conclusions

The halophilic *Bacillus tequilensis* was screened based on higher EPS production with good stability and an antagonistic effect against the growth of selected phytopathogens. Produced EPS was a heteropolymer made up of glucose, galactose, mannose, and glucuronic acid. It was amorphous, having excellent shear thinning and a pseudoplastic nature, and possessed a good water-holding capacity of 18% (18 g water/100 g EPS). The EPS was reported to have bioactive properties viz. antibacterial activity (the highest activity was obtained against *E. coli*), antioxidant activity, and anti-inflammatory activity. The deduced IC_50_ value of EPS was 174.21 µg/mL which was lesser than the standard anti-inflammatory drug (IC_50_ 186.17 µg/mL) which indicated high anti-inflammatory potential. Hence, the presented halophilic EPS was suitable to be used as coating material. The application of EPS coating on tomato fruits showed a significant reduction in weight loss (%) and maintaining the firmness of fruits which ultimately prolonged the shelf life of fruits. The comparative analysis revealed that EPS had a better shelf-life-prolonging capacity than uncoated as well as chitosan-treated tomatoes at both 4 °C and 30 °C whilst the outcome was comparable to standard xanthan gum. EPS showed maximum growth inhibition of the phytopathogenic fungus *Alternaria solani* and reduced the % disease incidence by 89.37% compared to uncoated fruits. Consequently, produced marine EPS was suitable for application as a coating material to prevent postharvest losses due to the onset of phytopathogens and was also reported to prolong the shelf life of coated fruits. 

## Figures and Tables

**Table 1:** Literature review of findings of applications of EPSs produced by halophilic bacteria.

Halophilic Bacteria	EPS Properties	Application	Reference
*Bacillus altitudinis*	Antimicrobial and antioxidant activity	Antagonism of pathogenic *Pseudomonas arugenosa*	[6]
*Pseudomonas stuzeri*	Antibiofilm forming and anti-biofouling potential	Treatment of antibiotic resistant infections and pervert biofouling in marine environment	[7]
*Alteromonas* sp.	Heavy metal binding capacity	Heavy metal remediation from polluted sites	[8]
*Geobacillus*, *B. licheniformis*	Antiviral and immune stimulatory actions	Therapy of viral infections viz. herpes	[9]
*Halomonas maura*	Electrospnning potential	Biomedical sector for sustained bioimaging, drug delivery via nano particles	[10]
*Halomonas alkaliantarctica*	High emulsification capability	Oil recovery	[11]
*Vagococcus carniphilus*	Potent flocculating activity comparable to xanthan gum	Industrial waste water flocculants	[12]
*Halomonas xianhensis*	Anticancer activity	Biomedical applications	[13]
Marine *Bacillus cereus* and *Brachybacterium* sp.	Antimicrobial activity	Potential use as antimicrobial agent	[14]
*Halomonas malpeensis YU-PRIM-29*	Emulsification activity, antioxidant activity	Potential petroleum hydrocarbon emulsification	[15]
Marine cynobacteria	Acidic nature and presence of uronic acid	Potential use in inhibiting the growth of toxic cyanobacteria in aquatic environment	[16]
*Halomonas strains*	High viscosity, Fucose present, Pseudoplastic behavior	Potential use in food industry for food preservation	[17]

**Table 2:** (a) Primary screening of marine samples for halophilic EPS producing bacteria. (b) Determination of growth inhibitory effect of EPS by agar diffusion assay.

(a)				
Isolate	EPS Content (g/L)	Optical Density	Dry Cell Mass (g/L)	
SWIS01	5.69 ± 0.27	0.84 ± 0.02	0.93 ± 0.04	
SWIS02	13.76 ± 0.25	0.99 ± 0.01	0.86 ± 0.02	
SSIS01	3.88 ± 0.26	1.05 ± 0.13	0.97 ± 0.13	
SSIS02	9.64 ± 0.37	1.83 ± 0.02	0.69 ± 0.10	
KSIS01	5.68 ± 0.33	1.04 ± 0.17	0.87 ± 0.09	
KSIS02	11.04 ± 0.26	1.04 ± 0.05	0.87 ± 0.02	
KWIS01	3.13 ± 0.27	1.17 ± 0.18	0.85 ± 0.12	
KWIS02	13.68 ± 0.34	2.02 ± 0.03	0.52 ± 0.12	
TSIS01	12.84 ± 0.18	0.87 ± 0.02	0.83 ± 0.02	
TSIS02	6.17 ± 0.29	0.96 ± 0.04	0.70 ± 0.03	
TWIS01	4.83 ± 0.27	0.68 ± 0.05	0.61 ± 0.03	
UWIS01	3.88 ± 0.39	1.45 ± 0.19	0.75 ± 0.07	
UWIS02	5.58 ± 0.54	1.00 ± 0.09	0.84 ± 0.06	
UWIS03	8.14 ± 0.36	1.18 ± 0.12	0.82 ± 0.05	
UWIS04	11.57 ± 0.29	0.78 ± 0.02	0.86 ± 0.02	
DWIS01	2.35 ± 0.62	0.85 ± 0.06	0.57 ± 0.10	
DWIS02	4.07 ± 0.29	0.74 ± 0.06	0.61 ± 0.04	
DWIS03	10.28 ± 1.18	1.57 ± 0.01	0.69 ± 0.02	
DUIS01	4.44 ± 0.41	1.46 ± 0.25	0.66 ± 0.04	
KAIS01	3.55 ± 0.61	1.30 ± 0.27	0.64 ± 0.04	
KAIS02	6.84 ± 0.58	1.31 ± 0.33	0.74 ± 0.07	
KEIS01	3.40 ± 0.66	1.03 ± 0.25	0.52 ± 0.11	
**(b)**				
**Halophilic EPS**	**Zone of Inhibition (mm) against Selected Phytopathogens**			
	***F. oxysporum***	***F. solani***	***A. solani***	***X. citri***
SWIS02	12.3 ± 1.09	11.82 ± 1.19	12.74 ± 0.85	11.8 ± 1.16
TSIS01	16.44 ± 1.48	18.32 ± 1.10	23.78 ± 1.25	21.04 ± 1.59
KSIS02	11.56 ± 0.83	12.20 ± 0.47	14.08 ± 1.35	14.76 ± 1.18
KWIS02	10.78 ± 0.43	12.50 ± 1.15	15.32 ± 0.69	14.46 ± 1.02
UWIS04	13.32 ± 1.22	14.56 ± 0.59	15.52 ± 1.15	18.18 ± 1.45
Antimicrobial agent	18.3 ± 0.95	20.04 ± 1.28	25.34 ± 1.15	22.8 ± 1.37

**Table 3:** Optimization parameters analyzed for higher EPS production by TSIS01.

Parameter	Optimum Condition
Media	Minimal media
Carbon source	Starch
Nitrogen source	Beef extract
Content of carbon source	25 g/L
Content of nitrogen source	3 g/L
K_2_HPO_4_ concentration	6 g/L
MgSO_4_ concentration	1 g/L
NaCl concentration	7%
Temperature	30 °C
pH	7.0
Incubation time	72–144 h
Static or shaking conditions	Shaking at 150 rpm
Water (D/W or sea water)	Sea water

**Table 4:** Effect of EPS coating on tomato fruits’ weight loss (%) and firmness coefficients of reaction constants K_c_ and R^2^ for first, second, and zero-order reactions at temperature treatments 4 °C and 30 °C.

% Weight Loss	T (°C)	*n* = 0			*n* = 1			*n* = 2		
		R^2^	K_c_	A_0_	R^2^	K_c_	A_0_	R^2^	K_c_	A_0_
UC	4	0.987	1.51	99.89	0.987	0.019	99.95	0.987	0.0003	100.0
	30	0.998	4.10	99.98	0.999	0.047	100.55	0.992	0.0002	100.0
EC	4	0.987	1.32	99.75	0.977	0.015	99.86	0.988	0.0006	102.0
	30	0.999	2.00	99.92	0.998	0.024	100.13	0.997	0.0004	100.0
CC	4	0.988	1.40	99.69	0.989	0.017	99.89	0.996	0.0005	100.0
	30	0.998	3.25	99.82	0.997	0.039	100.47	0.994	0.0003	100.0
XC	4	0.988	1.36	99.78	0.977	0.017	99.85	0.997	0.0006	100.0
	30	0.998	2.20	99.91	0.997	0.023	100.15	0.995	0.0004	100.0
**Firmness**	**T °C**	***n* = 0**			***n* = 1**			***n* = 2**		
		**R^2^**	**K_c_**	**A_0_**	**R^2^**	**K_c_**	**A_0_**	**R^2^**	**K_c_**	**A_0_**
UC	4	0.915	0.477	12.71	0.920	0.047	13.34	0.920	0.0051	12.45
	30	0.826	0.682	11.74	0.876	0.071	12.73	0.911	0.0070	11.75
EC	4	0.954	0.192	12.71	0.932	0.019	13.74	0.932	0.0020	12.91
	30	0.895	0.453	12.19	0.922	0.052	14.00	0.923	0.0050	12.17
CC	4	0.945	0.188	12.71	0.929	0.028	13.57	0.925	0.0030	12.81
	30	0.879	0.436	12.00	0.913	0.064	13.89	0.918	0.0065	11.98
XC	4	0.957	0.194	12.71	0.935	0.021	13.79	0.934	0.0020	12.87
	30	0.890	0.457	12.73	0.919	0.050	13.98	0.921	0.0055	12.14

**Table 5:** Effect of EPS coating on the shelf life of tomato fruits according to the deterioration kinetics models.

Treatment	4 °C		30 °C	
	Weight Loss	Firmness	Weight Loss	Firmness
UC	5	13	3	8
EC	17	35	13	25
CC	13	29	10	16
XC	18	37	12	22

**Table 6:** Marine sample collection sites.

Location	Coordinates
Sambhar lake	(26.9456° N, 75.2097° E)
Kutch	(23.7337° N, 69.8597° E)
Tithal	(20.5890° N, 72.9031° E)
Khambhat	(22.3181° N, 72.6190° E)
Udvada	(20.4863° N, 72.8722° E)
Ubharat	(21.0126° N, 72.7348° E)
Dumas	(21.0949° N, 72.7114° E)

**Table 7:** Treatments of tomato fruits.

Treatment	Composition	No. of Replication	Total Number of Tomatoes Treated
Experimental coating	EPS of *Bacillus tequilensis* (1%) + 1% glycerol + 0.5% Tween 80 + 1% Oleic acid	5	250
Positive control 1	Xanthan gum (1%) + 1% glycerol + 0.5% Tween 80 + 1% Oleic acid	5	250
Positive control 2	Chitosan (1%) + 1% glycerol + 0.5% Tween 80 + 1% Oleic acid	5	250
Negative control (Uncoated)	Uncoated tomatoes (immersed in D/W)	5	250

## Data Availability

Data are contained within the article.
